# Evaluation of Clinical Outcomes in Dogs with Malignant Intranasal Tumors Treated with Radiotherapy: A Retrospective Study of 40 Cases

**DOI:** 10.3390/cancers18122013

**Published:** 2026-06-22

**Authors:** Simone Carvalho dos Santos Cunha, Bianca Moreira Angelim, Rebeca Herdade, Karen Cristina de Souza da Rocha Dias, Laís Calazans Menescal Linhares, Rafael Costa Bitencourt, Guilherme Andraus Bispo, Felipe Noleto de Paiva, Andrigo Barboza de Nardi

**Affiliations:** 1 Vet Radioterapia, Av. das Américas, 505, Loja A—Barra da Tijuca, Rio de Janeiro 22631-000, RJ, Brazil; simonecsc@gmail.com (S.C.d.S.C.); vet.radio.sp@gmail.com (B.M.A.); rebecaherdade@hotmail.com (R.H.); karencristinasr@icloud.com (K.C.d.S.d.R.D.); andrigo.barboza@unesp.br (A.B.d.N.); 2 Department of Veterinary Clinic and Surgery, School of Agricultural and Veterinary Sciences, São Paulo State University, Via de Acesso Professor Paulo Donato Castellane S/N—Vila Industrial, Jaboticabal 14884-900, SP, Brazil; rafael.c.bitencourt@unesp.br; 3 Department of Clinic, Surgery, and Animal Reproduction, School of Veterinary Medicine, São Paulo State University, R. Clóvis Pestana, 793—Dona Amélia, Aracatuba 16050-680, SP, Brazil; g.bispo@unesp.br; 4 Centro Oncológico Veterinário Goiás (COV-GO), R. C-235, 340—Jardim América, Goiania 74290-050, GO, Brazil; n-paiva@hotmail.com

**Keywords:** canine, cobalt-60, nasal neoplasia, radiation therapy, prognostic factors

## Abstract

Background/Objectives: Intranasal tumors are common malignancies in dogs, characterized by locally aggressive behavior and clinical signs such as epistaxis, nasal discharge, and facial deformity. Radiotherapy (RT) is considered the treatment of choice due to anatomical limitations to surgical resection. This study aimed to evaluate clinical outcomes, toxicity, and prognostic factors in dogs with primary malignant intranasal tumors treated with cobalt-60–based megavoltage radiotherapy. Methods: This retrospective study included 40 dogs with histopathologically confirmed primary malignant intranasal tumors treated between September 2018 and February 2025 at a veterinary radiotherapy clinic in Rio de Janeiro, Brazil. Medical records were reviewed for patient demographics, tumor characteristics, treatment protocols, response, toxicity, and survival outcomes. Tumors were staged using modified Adams criteria based on computed tomography. Definitive-intent protocols (*n* = 32) delivered 48–54 Gy in 10–13 fractions administered three to five times weekly, while palliative protocols consisted of either four fractions of 8 Gy delivered once weekly or five fractions of 4 Gy delivered daily. Results: Adenocarcinoma was the most common histologic subtype (42.5%), and 82.5% of dogs had stage III–IV disease. The objective response rate was 82.5% (CR: 17.5%; PR: 65.0%), with clinical benefit observed in 92.5% of cases. Acute toxicity was frequent but manageable, primarily affecting skin, oral mucosa, and eyes. Overall median progression-free interval (PFI) and survival time (MST) were 382 days and 430 days, respectively. Stage IV disease was significantly associated with shorter survival when compared to stage I-III (MST 345 vs. 1063 days, respectively; *p* = 0.016). Treatment response was significantly associated with PFI in univariate analysis (*p* < 0.05). Conclusions: Radiotherapy provided high response rates and meaningful clinical benefit with acceptable toxicity in dogs with malignant intranasal tumors, highlighting the importance of early diagnosis and treatment. Further prospective studies with standardized protocols are warranted.

## Simple Summary

Intranasal tumors are common cancers in dogs, frequently causing bleeding, nasal discharge, and facial deformity. Because these tumors are located near critical structures, surgery is often not feasible, making radiotherapy the primary treatment option. This study reviewed medical records of 40 dogs with malignant intranasal tumors treated with cobalt-60 radiotherapy at a veterinary clinic in Brazil. Most dogs had advanced disease at presentation. Radiotherapy resulted in tumor shrinkage or stabilization in the majority of cases, with improvement of clinical signs in most dogs. Side effects were common but manageable, primarily affecting the skin, oral mucosa, and eyes. Dogs with more advanced tumors had shorter survival times, highlighting the importance of early diagnosis and treatment. These findings suggest that radiotherapy provides meaningful clinical benefit with acceptable toxicity and may help guide treatment decisions for dogs with intranasal cancer.

## 1. Introduction

Malignant intranasal tumors in dogs represent relatively uncommon neoplasms, accounting for approximately 1–2% of all canine tumors [1]. The affected population typically consists of older dogs, with a mean age at diagnosis ranging from 9 to 11 years, with higher prevalence in dolichocephalic and mesocephalic breeds [2].

Approximately two-thirds are epithelial in origin, predominantly adenocarcinomas and undifferentiated carcinomas, while the remaining third comprises mesenchymal tumors [1,2,3]. These tumors are characterized by aggressive local invasion with extension into adjacent structures, while distant metastasis occurs relatively late in the disease course, reported in approximately 6–13% of cases at initial diagnosis [3]. Clinical presentation includes nasal discharge, epistaxis, sneezing, and respiratory stertor, with disease progression leading to facial deformity, exophthalmos, and neurological signs when intracranial invasion occurs. Without treatment, median survival times remain only 3–6 months, primarily due to progressive local disease [4].

Although surgery and systemic therapies may be considered in selected cases, complete surgical excision is often challenging because of the complex anatomy of the nasal cavity and the locally invasive behavior of intranasal tumors, while systemic therapies used as sole treatment modalities generally provide limited local tumor control [3,4]. Radiotherapy (RT) also has limitations, including the need for multiple anesthetic procedures, potential acute and late adverse effects, limited availability, and relatively high treatment costs. Nevertheless, RT remains the cornerstone of treatment for most dogs with malignant intranasal tumors, with reported median survival times (MST) ranging from 7 to 20 months following definitive-intent protocols [1,3,5].

Modern RT has evolved from orthovoltage units to megavoltage radiation sources, including cobalt-60 units and linear accelerators, enabling techniques such as three-dimensional conformal radiotherapy (3D-CRT) and intensity-modulated radiotherapy (IMRT) [6].

The characterization of prognostic factors has yielded variable results across studies. Some investigations have identified cribriform plate invasion, tumor volume, and histologic subtype as potential prognostic indicators [1,2,3], while other studies have failed to confirm these [5]. Questions remain regarding optimal fractionation schedules, the impact of tumor stage on outcomes, and the relationship between radiologic tumor response and long-term survival [3,4,5,6,7]. Treatment-related toxicity, including cutaneous reactions, mucositis, and ocular complications, remains a significant consideration potentially impacting quality of life [6].

The present study aims to provide a detailed retrospective analysis of clinical outcomes, prognostic factors, and toxicity in 40 dogs with malignant intranasal tumors treated with megavoltage three-dimensional conformal RT at a Brazilian radiotherapy veterinary clinic.

## 2. Materials and Methods

### 2.1. Case Selection

This retrospective observational study included 40 dogs diagnosed with primary malignant intranasal tumors that underwent RT between September 2018 and February 2025 at Vet Radioterapia clinic (Rio de Janeiro, Brazil). The present study represents an updated retrospective evaluation of dogs treated at the same institution [8]. A small subset of cases (*n* = 4) was previously included in the earlier report.

A consecutive sampling approach was used, whereby all dogs meeting the inclusion criteria during the study period were eligible for inclusion. Dogs were included if they had a histopathologically confirmed primary malignant intranasal tumor treated with megavoltage radiotherapy (RT). Dogs were excluded if they were clinically unstable for multiple anesthetic procedures, had secondary nasal cavity involvement by tumors of extra-nasal origin, were diagnosed with benign tumors, or died prior to treatment response assessment by clinical evaluation or imaging.

### 2.2. Procedures

Data collection from medical records included the following variables: patient demographics (breed, sex, and age); tumor characteristics (tumor location, tumor dimensions, histological subtype, clinical signs, and tumor stage based on computed tomography [CT]); treatment-related data (treatment dates, radiotherapy protocol including fractionation, total dose, and number of sessions, as well as adjuvant treatments such as chemotherapy, targeted therapies, or surgery); treatment response and toxicity (clinical and/or imaging-based response and adverse effects); and outcome measures, including disease-free interval (DFI), progression-free interval (PFI), survival time (ST), and follow-up status. Staging was performed according to modified Adams’ tumor staging [9], using CT imaging of the skull.

Radiotherapy protocols were classified as definitive-intent or palliative. Definitive-intent protocols were prescribed with the goal of achieving durable local tumor control, whereas palliative protocols were intended primarily to alleviate clinical signs and improve quality of life in dogs with advanced disease or relevant comorbidities.

All treatments were delivered using a Theratron 780C (Cobalt-60) unit (Best Theratronics, Kanata, ON, Canada). Definitive radiotherapy protocols consisted of 10 to 13 fractions administered three to five times per week, for a total dose of 48 to 54 Gy, with fraction doses ranging from 3 to 5 Gy. Palliative regimens were employed in selected cases, including dogs with advanced tumors, cribriform plate invasion associated with neurological signs, or severe comorbidities that precluded definitive treatment. The palliative protocol consisted of four fractions of 8 Gy delivered once weekly or five fractions of 4 Gy delivered daily.

RT planning was based on axial CT images using a three-dimensional conformal planning system (CAT3D or MIRS). Gross tumor volume (GTV) was defined as the visible tumor identified on CT imaging. Planning target volume (PTV) encompassed the CTV with a further margin of 1 cm. Organs at risk (OAR) routinely contoured and evaluated included the eyes, mandible and brain.

Supportive care measures were routinely implemented to minimize treatment-related toxicity. Dogs receiving irradiation involving the ocular region underwent ophthalmologic evaluation before treatment and received preventive ocular lubrication and corneal protection when indicated. Oral mucositis was managed with topical therapies and laser treatment as needed, while prophylactic topical products were recommended to minimize radiation-associated skin reactions.

Radiologic tumor response (CT-response) was assessed according to the Veterinary Cooperative Oncology Group response evaluation criteria (cRECIST v1.0), based on post-treatment CT scans when available. Responses were classified as complete response (CR), partial response (PR), stable disease (SD), or progressive disease (PD). For dogs that did not undergo follow-up CT imaging, tumor response was assessed clinically based on improvement, stabilization, or worsening of nasal signs and categorized using analogous terminology (clinical CR, PR, SD, or PD). For the purposes of this study, clinical benefit was defined as achieving CR, PR, or SD, whereas dogs with PD were considered non-responders.

### 2.3. Statistical Analysis

Median survival time (MST) was defined as the time from the first RT session to death. Dogs that were alive at the end of the study period or lost to follow-up were censored at the date of last contact. Deaths attributed to tumor progression, metastatic disease, or clinical deterioration related to the tumor were considered events for survival analysis. Progression-free interval (PFI) was calculated from the first RT session to the date of documented disease progression, either local recurrence or metastasis. Progression was confirmed by CT or suspected based on relapse or worsening of clinical signs in the absence of follow-up imaging. Dogs that remained alive without evidence of progression or died of unrelated causes were censored at the date of last follow-up. Disease-free interval (DFI) was defined as the time from completion of RT to local recurrence, metastasis, or tumor-related death. Only dogs that achieved a complete response (CR) were included in this analysis. Dogs without evidence of recurrence at the end of the study period or that died of unrelated causes were censored at the date of last follow-up. CT-based response was assessed only in dogs with at least one follow-up CT scan. Overall response included both CT-based and clinical assessments of response.

Several factors were evaluated for potential associations with PFI and MST. These included age, body weight, sex (male/female), epistaxis, RT protocol (definitive vs. hypofractionated), and adjuvant treatment (yes/no). Tumor-related variables included CT-based response, overall response (CT-based response plus clinical response), GTV and tumor stage (stage 1–3 and stage 4). Survival outcomes and associations between categorical clinical variables were evaluated using complementary statistical approaches.

Categorical comparisons were performed using Fisher’s exact test to assess proportional differences between groups. Time-to-event outcomes were analyzed using Kaplan–Meier survival curves, with group comparisons conducted via the log-rank test. Prognostic effects of continuous and categorical variables on outcomes of interest were assessed using univariate Cox proportional hazards models, with hazard ratios (HRs) and corresponding 95% confidence intervals (95% CIs) reported. Variables included in the multivariable analysis were selected based on clinical relevance and results from the univariable models. The proportional hazards assumption was tested using Schoenfeld residuals. A significance level of *p* < 0.05 was adopted for all tests. All statistical analyses were performed using R software (version 4.3.3; R Core Team, 2024).

## 3. Results

### 3.1. Study Population

A total of 40 dogs with malignant intranasal tumors treated with RT were included. Mixed-breed dogs were the most frequent breed (*n* = 16), followed by Shih-tzu (*n* = 7). Other breeds included German Spitz, Beagle, Yorkshire Terrier, Bulldog, Boxer, Dalmatian, Rottweiler, Dachshund, Pit Bull, Husky, Poodle, American Staffordshire Terrier, Scottish Terrier, French Bulldog, and Labrador Retriever. The mean age at diagnosis was 11.2 years (median 11, range 4–19 years). Of these, 26 (65%) were female and 19 (35%) were male. A summary of patient demographics is presented in Table 1.

All dogs presented with clinical signs related to the intranasal tumor before radiotherapy. The most common presenting signs included epistaxis (*n* = 24) and sneezing (*n* = 19). Other frequently observed signs were nasal discharge (*n* = 11), facial deformity (*n* = 5), and seizures (*n* = 2).

### 3.2. Tumor Features and Staging

Information regarding histopathology was obtained for all cases. Tumor types were adenocarcinoma (*n* = 17), unspecified carcinoma (*n*= 6), transitional cell carcinoma (*n* = 2), fibrosarcoma (*n* = 2), osteosarcoma (*n* = 1), chondrosarcoma (*n* = 2), neuroendrocrine tumor/olfactory neuroblastoma (*n* = 1), unspecified sarcoma (*n* = 3), malignant odontogenic tumor (*n* = 1) and intranasal neoplasia of unknown origin (*n* = 3) (Table 1).

Modified Adams’ staging of dogs at the time of planning CT was as follows: stage 1 (*n* = 2), stage 2 (*n* = 5), stage 3 (*n* = 16), and stage 4 (*n* = 17). Among the stage 4 cases, 13 dogs presented with less than full-thickness cribriform plate erosion, while four dogs exhibited intracranial tumor extension.

Tumor volume analysis was performed in dogs for which complete three-dimensional CT measurements were available (*n* = 30). The median intranasal tumor volume was 45.8 cm^3^ (mean, 96.2 cm^3^; range, 2.9–281.2 cm^3^), based on measurements obtained from pre-treatment CT scans. Ten dogs were excluded from this analysis due to the unavailability of CT measurements.

Thoracic radiographs were performed at the time of initial diagnosis in all dogs, with no evidence of distant metastasis observed. Regional lymph node evaluation was not routinely performed. During follow-up, metastatic disease was diagnosed in three dogs based on diagnostic imaging findings, with cytological confirmation obtained when available. One dog developed pulmonary and mandibular lymph node metastases shortly after completion of RT, one developed pulmonary metastases 30 days after RT, and one developed pulmonary and skeletal metastases 120 days after RT. The skeletal metastases involved the right ischium and left femur and were identified by computed tomography.

### 3.3. Radiotherapy Protocol

Definitive-intent protocols were the most frequently employed (*n* = 32), with the majority receiving 12 × 4.2 Gy three times per week (*n* = 17). Less common regimens included 18 × 3 Gy (*n* = 5), 13 × 3.8 Gy (*n* = 4), 12 × 4 Gy (*n* = 3), 15 × 3.5 Gy (*n* = 2) and 10 × 5 Gy (*n* = 1). Palliative protocols were used in eight dogs, most commonly 4 × 8 Gy delivered once weekly (*n* = 7), and in one dog, a 5 × 4 Gy protocol was delivered daily. Among these dogs, seven were in stage 4, with cribriform plate invasion. One dog was in stage 3, but the owner refused the definitive protocol due to time restrictions.

Information on acute adverse events was available for 37 of the 40 dogs included in the study. Toxicities were graded according to the most recent ACVR/ECVDI consensus guidelines published by Poirier et al. [9] (Table 2). Grade 1 and grade 2 toxicities were the most frequently observed, while grade 3 and grade 4 were less common and confined to tissues within the radiation field.

Cutaneous toxicity was the most common occurring in 34 dogs (91.9%). Moist desquamation was observed in 16 of 37 dogs (43.2%), whereas 11 dogs (29.7%) developed dry desquamation and two exhibited alopecia only. Information regarding the type of desquamation was unavailable for five dogs. Mucositis occurred in 20 dogs (54%), and was classified as grade 3 in nine dogs, mostly due to the presence of superficial ulcers. Ocular toxicity occurred in 19 dogs (51.3%) and ranged from mild discharge (grade 1, *n* = 5) to superficial corneal ulcers (grade 3, *n* = 7) or a perforated ulcer (grade 4, *n* = 1). Other ocular signs included mild to intense blepharospasm (*n* = 4), conjunctivitis (*n* = 1), and keratitis (*n* = 1). Neurologic toxicity was uncommon, with only one dog experiencing an isolated grade 2 seizure.

The time from the first radiotherapy session to the onset of the first acute adverse event was available for 26 dogs. The median time to toxicity onset was 13.5 days (range, 5–25 days; mean, 14.8 ± 5.7 days). Most adverse events emerged between 10 and 20 days after treatment initiation, consistent with the expected time course of acute radiation reactions.

Late adverse effects were documented in seven dogs. Among these, late adverse events were mild and restricted to cutaneous and ocular changes. One dog developed a cataract (grade not assessed), and three dogs showed mild late skin changes classified as VRTOG grade 1, including alopecia, leukotrichia, and pigmentation changes. No moderate or severe late toxicities (VRTOG grades 2–4) were identified in this subset.

### 3.4. Response Evaluation

Follow-up CT imaging after RT was performed in 31 of 40 dogs. Of these, 24 (77.4%) underwent only one follow-up scan and seven (22.6%) had two scans. The exact date of the first follow-up CT was available for 29 of the 31 dogs, and for three of the seven dogs with two scans. The median interval between the last RT session and the first follow-up CT was 53 days (mean, 53 days; range, 11–95 days). The intervals between the first and second follow-up CT were 100, 132, and 166 days for each of the three dogs, respectively.

CT response evaluation was available for the 31 dogs that underwent follow-up CT imaging. Nineteen dogs (61.3%) achieved a PR, six (19.3%) a CR, four (12.9%) SD, and three (9.6%) exhibited PD. Among the dogs classified as PR, the median reduction in tumor volume was 76% (range, 41–97%), with 40% exhibiting > 80% reduction and 27% achieving ≥ 90%.

For the nine dogs for whom follow-up CT was not available, clinical response was assessed based on improvement or worsening of presenting signs, including nasal discharge, epistaxis, facial deformity, and neurological symptoms. Among them, seven dogs were clinically classified as PR, one as CR, and one as SD. Overall, combining CT and clinical assessments, responses included 26 PR, 7 CR, 5 SD, and 3 PD, yielding an objective response rate of 82.5%.

Overall clinical benefit was achieved in 37 of 40 dogs (92.5%), consisting of CR, PR, or SD. Although the three dogs (7.5%) classified as PD exhibited transient symptomatic improvement immediately after RT, clinical benefit was defined exclusively based on CT response whenever imaging was available. Therefore, dogs with PD were considered non-responders regardless of any temporary clinical improvement. 

### 3.5. Adjuvant Therapies After RT

A total of 31 dogs received adjuvant antitumor therapy after completion of RT, while nine dogs were managed with RT alone. Toceranib phosphate (Palladia^®^; Zoetis Inc., Kalamazoo, MI, USA) was the most commonly used agent, administered to 26 dogs, alone or in combination with other therapies. Other drugs included conventional chemotherapy (*n* = 8), firocoxib (*n* = 3), and rapamycin (*n* = 1).

Among the dogs treated with both toceranib phosphate and chemotherapy (*n* = 5), cytotoxic chemotherapy was initiated only after discontinuation of toceranib phosphate. Three dogs received toceranib phosphate in combination with firocoxib. Of the eight dogs that underwent cytotoxic chemotherapy, two received carboplatin alone, two received doxorubicin alone (both after toceranib phosphate discontinuation), one received alternating carboplatin and doxorubicin, and three received metronomic cyclophosphamide as a sole agent, two of which had also previously received toceranib phosphate.

### 3.6. Outcome Data

Overall MST was 430 days (95% confidence interval [CI]: 327—not estimable). At the end of the study period, 19 dogs were still alive and were censored at their last follow-up, while four dogs were lost to follow-up at 35, 75, 94, and 138 days. Among the dogs still alive, the median follow-up time was 252 days (range, 123–850 days).

The median PFI was 382 days (95% CI: 175—not estimable). Twenty-one dogs were censored: two died at 1063 and 1771 days without clinical evidence of tumor progression, 13 were alive without evidence of progression, and four were lost to follow-up at 35, 75, 94, and 138 days.

DFI was calculated for the six dogs with CR, with a median of 465 days (mean 649 days, range 57–1771 days). Of these, three dogs showed no evidence of recurrence at the end of the study period: two died without tumor relapse (DFI 1063 and 1771 days), and one remained alive and disease-free (DFI 181 days). Two dogs (40%) experienced tumor recurrence: one died from local tumor progression (DFI 78 days, ST 152 days), and one was alive with recurrent disease (DFI and ST 749 days). The remaining dog was lost to follow-up at 57 days. Overall, the majority (60%) of dogs maintained CR for over one year.

Nineteen dogs (47.5%) were alive at the end of the study period. Of these, 14 (73.7%) remained free of disease progression, while five (26.3%) experienced tumor recurrence or progression but were still alive at the time of data collection. In terms of overall response, 14 dogs (73.7%) achieved a PR, two (10.5%) a CR, two (10.5%) an SD, and one (5.3%) a PD. Among these 19 dogs, only one had its response assessed clinically and was classified as PR.

Among the 16 dogs (40%) that died during the study, 13 deaths (81.2%) were attributed to tumor progression or recurrence. For these dogs, median PFI and ST were 164 days (mean 179, range 15–569) and 207 days (mean 275, range 143–653), respectively.

### 3.7. Survival Analysis

**Figure 1:**
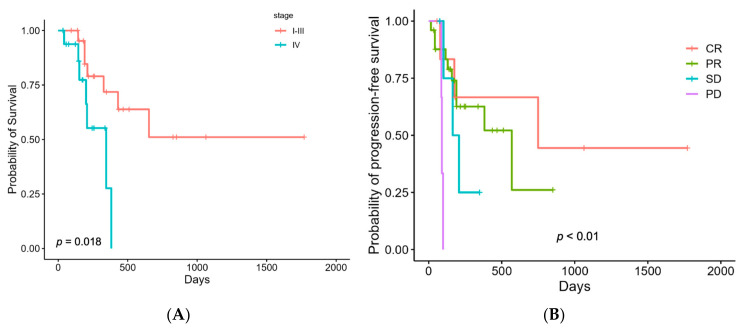
Kaplan–Meier curves for survival outcomes. (**A**) Overall survival time (ST) stratified by tumor stage (I–III vs. IV). (**B**) Progression-free interval (PFI) stratified by overall response (CR, PR, SD, PD).

In the univariate Cox analysis, tumor stage (I–III vs. IV) was the only variable significantly associated with overall survival (Table 3). The MST was 1063 days (95% CI: 430—not estimable) for dogs with stage I–III tumors and 345 days (95% CI: 200—not estimable) for those with stage IV tumors. Dogs with stage IV disease had a nearly fourfold increased risk of death compared to those in stages I–III (HR = 3.78; *p* = 0.027), as illustrated by Kaplan–Meier survival curves (Figure 1A).

Regarding histological diagnosis, the MST was 653 days (95% CI: 210—not estimable) in the 26 dogs with nasal carcinoma and 430 days (95% CI: 210—not estimable) in the eight dogs with nasal sarcoma; however, this difference was not statistically significant (*p* = 0.98).

Regarding protocol type, the MST was 653 days (95% CI: 327—not estimable) for dogs treated with definitive protocols and 291 days (95% CI: 152—not estimable) for those treated with hypofractionated protocols, with a median PFI of 569 days (95% CI: 190—not estimable) and 166 days (95% CI: 130—not estimable), respectively. Although hypofractionation was associated with trends toward increased risks of both death and progression (HR = 2.74 and HR = 2.18, respectively), neither difference was statistically significant (Table 3).

For PFI, both CT-based and overall response were significantly associated (*p* = 0.01 and *p* = 0.02, respectively), with Kaplan–Meier curves confirming a higher risk of progression in dogs with PD compared to those with CR (*p* = 0.009 and *p* = 0.006) (Table 3, Figure 1B).

**Figure 2:**
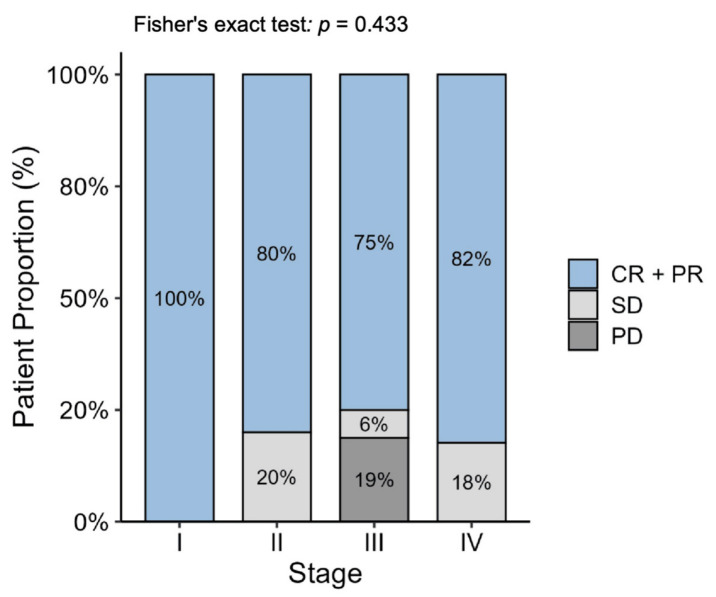
Distribution of overall response according to clinical stage. Advanced-stage disease (stage IV) was not significantly associated with poorer treatment response.

As demonstrated in Figure 2, the distribution of overall response appeared comparable across clinical stages, with no consistent pattern indicating diminished response in more advanced disease. Statistical evaluation corroborated this observation, as no significant association was identified between these variables (Fisher’s exact test, *p* = 0.667).

In the multivariate Cox model for survival time, which included clinical stage, RT protocol, adjuvant therapy, and CT-based response, none of the variables remained statistically significant predictors of outcome (Table 4). Interpretation was limited by substantial case loss and a very low number of events (*n* = 7), resulting in wide confidence intervals. Although PD showed the strongest trend toward inferior survival (HR = 10.33), this association did not reach statistical significance. Similarly, in the multivariate Cox model for PFI, which included clinical stage, RT protocol, and adjuvant therapy, none of the variables were significantly associated with time to progression (global test, *p* = 0.40) (Table 4). Overall, the multivariate analyses did not identify independent predictors of either survival time or PFI in this cohort.

## 4. Discussion

This study evaluated clinical outcomes and toxicity in 40 dogs with malignant intranasal tumors treated with RT using a cobalt-60 unit. Median PFI and MST were 382 and 430 days, respectively. These results are consistent with previous studies evaluating RT in canine intranasal neoplasia, which have reported MSTs ranging from 240 to 488 days using different radiotherapy platforms, including cobalt-60–based RT and linear accelerator (LINAC)-based megavoltage RT [3,8,11,12,13,14]. However, direct comparisons across studies are limited by substantial heterogeneity in case selection, stage distribution, and treatment protocols. For example, Morgan et al. [15] reported an MST of 435 days in dogs treated with cytoreductive surgery followed by definitive fractionated LINAC-based megavoltage RT, whereas Iseri et al. [14] described a MST of 488 days using megavoltage RT in a cohort in which only 16.6% of dogs had stage IV disease. In the present study, comparable survival times were achieved despite a relatively high proportion of dogs with advanced disease (42.5% stage IV) and most dogs (87.5%) received RT as sole therapy.

Unlike most contemporary RT studies, which primarily included definitive-intent protocols [3,14,15], our cohort included eight dogs (20%) treated with palliative hypofractionated protocols. Although hypofractionation did not significantly affect MST or PFI, trends toward higher risks of death and progression were observed. The inclusion of these palliative cases likely contributed to the comparatively shorter MST observed in dogs receiving hypofractionated protocols compared to definitive ones (291 vs. 653 days, respectively). However, these findings should be interpreted with caution, as treatment allocation was based on clinical considerations rather than random assignment, and dogs receiving palliative protocols generally had more advanced disease or comorbidities.

Tumor stage (I–III vs. IV) was the only factor significantly associated with survival in our study, reinforcing previous evidence that cribriform plate destruction (stage IV) is a strong negative prognostic indicator [3,8,9,16]. Although dogs with stage IV disease had significantly shorter survival compared to those with stage I–III tumors (MST 345 vs. 1063 days), the MST achieved in these cases was longer than that reported in earlier studies involving advanced disease (132–274 days) [8,9,17,18]. In contrast, several other case series did not identify a significant association between tumor stage and survival [5,19,20,21,22], potentially due to differences in case selection, stage distribution, and RT protocols among studies.

In the present study, objective response was high, with 82.5% of dogs achieving PR or CR and 92.5% showing clinical benefit. These rates were similar to those obtained in previous studies, with up to 94% clinical benefit [3,8,14,15]. Response rates did not differ significantly across stages, consistent with findings from stereotactic radiation therapy (SRT) studies [23] and other conventional RT studies showing that early radiologic response is not strongly stage-dependent [15,24].

Epistaxis has been described as a negative prognostic factor in untreated intranasal carcinomas [25], but most RT studies do not confirm this association [15,22,26]. Similarly, despite a trend toward increased risk of shorter MST and PFI in our study (HR 2.5 and 2.9), epistaxis was also not statistically significant.

Pre-treatment tumor volume was not associated with CT-based or overall response, MST, or PFI, consistent with prior studies showing poor predictive value for absolute nasal tumor volume [7,26]. Only volume normalized to nasal cavity dimensions has shown prognostic significance [7], suggesting that relative rather than absolute tumor burden may better reflect clinical behavior.

Tumor histology may influence outcomes. Previous studies reported poorer responses and shorter survival for intranasal sarcomas compared with carcinomas [3,15]. Although our sarcoma sample was too small (*n* = 8) and unbalanced for meaningful statistical analysis, this remains a potential area for future investigation.

Adjuvant therapy was not associated with improved MST or PFI in this cohort, although interpretation is limited by non-standardized protocols and the high proportion of treated dogs (77.5%). Similar lack of survival benefit has been reported for chemotherapy [3,20], toceranib [27], and firocoxib [28]. It is important to note that some studies found improvement in symptoms with systemic therapy (firocoxib and toceranib phosphate), suggesting potential short-term clinical utility without clear survival impact [27,28].

Most dogs experienced only mild to moderate acute toxicity; however, many developed grade assignments in multiple tissues, resulting in a relatively high proportion of grade ≥ 3 events when assessed per tissue rather than per patient. Earlier megavoltage and orthovoltage RT studies reported substantial ocular morbidity, including frequent vision loss [11,12,29]. In contrast, high-grade ocular toxicity in our cohort was limited mostly to uncomplicated corneal ulcers, and all cases resolved with treatment. More recent conformal techniques (IMRT, SRT) typically report mostly low-grade acute reactions [23,30,31,32] and differences in case complexity, particularly the high prevalence of stage IV disease in our population, may explain the higher rates of grade ≥ 3 toxicity observed here. Acute toxicity among hypofractionated cases was mostly low-grade, consistent with previous reports [5,28].

Late toxicity was documented in seven dogs and was mild (VRTOG grade 1), consistent with recent conformal RT studies [8]. However, the true incidence of late adverse effects may have been underestimated because follow-up duration was limited for many patients. This limitation is particularly relevant given that most dogs (35/40) were treated during the final years of the study period (2024–2025), thereby reducing the opportunity for detection of late radiation-associated toxicities.

Metastatic disease occurred in 7.5% of dogs, aligning with prior reports (6–13%) [3,30]. In the present study, metastatic lesions were detected between 2 and 120 days after completion of RT, although the small number of events precludes further conclusions regarding metastatic behavior.

This study is limited by its retrospective design, relatively small sample size, heterogeneous tumor histologies, and the inclusion of both definitive and hypofractionated protocols, which may confound outcome interpretation. Follow-up imaging was inconsistent, limiting accurate assessment of CT-based response and late toxicity. Furthermore, response assessment in a subset of dogs was based on clinical evaluation rather than follow-up CT imaging, which may not fully reflect objective tumor response and should therefore be interpreted with caution. Staging was incomplete in some cases, and adjuvant systemic therapy was not standardized. Additionally, 26 of 40 dogs (65%) were censored at the time of analysis, reflecting that a substantial proportion of dogs were still alive, had not experienced disease progression, or died from non–tumor-related causes. This, in turn, reduced the precision of survival estimates and precluded reliable estimation of the upper limits of the confidence intervals.

## 5. Conclusions

In conclusion, RT provided high response rates and meaningful clinical benefits in dogs with malignant intranasal tumors. Survival outcomes were comparable to those reported in previous studies, despite the predominance of advanced-stage disease in this cohort. Advanced tumor staging and poor treatment response emerged as the strongest predictors of outcome, highlighting the importance of early diagnosis and treatment to improve clinical results. Future prospective studies are warranted to further elucidate the role of tumor biology, refine volumetric assessment methods, and evaluate the integration of systemic therapies and different radiotherapy protocols in achieving improved long-term disease control and survival.

## Figures and Tables

**Table 1:** Patient demographic data and tumor characteristics for 40 dogs treated with radiotherapy for malignant intranasal tumors.

Patient Characteristics	
Median (range) age (years)	11 (4–19)
Median (range) weight (kg)	14.8 (2.4–44)
Sex (No. [%] of dogs)	
Female	26 (65)
Male	14 (35)
Tumor characteristics (No. [%] of dogs)	
Histologic classification	
Adenocarcinoma	17 (42.5)
Carcinoma ^1^	8 (20)
Fibrosarcoma	2 (5)
Osteosarcoma	1 (2.5)
Chondrosarcoma	2 (5)
Unspecified sarcoma	3 (7.5)
Neuroendocrine tumor ^2^	1 (2.5)
Malignant odontogenic tumor	1 (2.5)
Intranasal neoplasia of unknown origin	4 (10)
Tumor stage ^3^	
T1	2 (5)
T2	5 (12.5)
T3	16 (40)
T4	17 (42.5)

^1^ Unspecified carcinoma (*n* = 6) and transitional cell caricnoma (*n* = 2); ^2^ suspected olphatory neuroblastoma; ^3^ tumor stage was assigned using a modified Adams system: T1—disease limited to nasal or sinus cavities without bone loss; T2—bony involvement without orbital or soft-tissue extension; T3—invasion of the orbit or adjacent soft tissues; T4—extension into the nasopharynx or across the cribriform plate.

**Table 2:** Acute radiotherapy-associated adverse events scored according to VRTOG acute toxicity criteria (grades 0–3).

VRTOG	Eyes	Skin	Mucous Membrane	CNS
Acute, Grade 0	0	0	0	0
Acute, Grade 1	5	13	5	0
Acute, Grade 2	3	4	5	0
Acute, Grade 3	10	9	9	0
Acute, Grade 4	1	3	2	0

Abbreviations: VRTOG, Veterinary Radiation Therapy Oncology Group; CNS, central nervous system. Values represent the number of dogs (*n* = 35) experiencing acute toxicity in each organ system and grade (0–4). Toxicities were scored following the 2023 ACVR/ECVDI consensus [10].

**Table 3:** Results of univariable analyses to identify variables associated with survival outcomes.

Univariable Analysis	*n*	Progression-Free Interval	Overall Survival
		*p*-Value	*p*-Value
Tumor stage		0.118	0.027
T1–T3	23		
T4	17		
Epistaxis		0.136	0.150
No	16		
Yes	24		
Tumor volume (pre-RT)		0.822	0.520
<45.8 cm^3^	15		
>45.8 cm^3^	15		
RT protocol		0.145	0.103
Definitive	32		
Hypofractionated	8		
Adjuvant therapy		0.663	0.254
No	9		
Yes	31		
CT response		0.010	0.400
CR	6		
PR	19		
SD	4		
PD	3		
Overall response (CT + clinical)		0.020	0.770
CR	7		
PR	26		
SD	5		
PD	3		

Abbreviations: RT, radiotherapy; CR, complete response; PR, partial response; SD, stable disease; PD, progressive disease.

**Table 4:** Results of multivariable Cox proportional hazards regression analyses identifying variables associated with progression-free survival and overall survival.

Multivariable Analysis	Progression-Free Interval		Overall Survival	
	HR (95% CI)	*p*-Value	HR (95% CI)	*p*-Value
Tumor stage T1–T3 vs. T4	1.85 (0.51–6.65)	0.348	2.81 (0.13–63.17)	0.51
RT protocol Definitive vs. Hypofractionated	1.45 (0.4–5.31)	0.572	3.31 (0.1–113.52)	0.51
Adjuvant therapy Yes vs. No	0.93 (0.24–3.59)	0.913	1.48 (0.14–15)	0.74
CT response				
PR vs. CR	-	-	1.47 (0.12–16.88)	0.76
SD vs. CR	-	-	2.55 (0.06–100.88)	0.62
PD vs. CR	-	-	10.33 (0.65–163.62)	0.097

Abbreviations: HR, hazard ratio; CI, confidence interval; RT, radiotherapy; CR, complete response; PR, partial response; SD, stable disease; PD, progressive disease.

## Data Availability

The original contributions presented in this study are included in the article. Further inquiries can be directed to the corresponding author.

## Abbreviations

The following abbreviations are used in this manuscript:

RT	Radiotherapy
CT	Computed tomography
CR	Complete response
PR	Partial response
SD	Stable disease
PD	Progressive disease
MST	Median survival time
PFI	Progression-free interval
DFI	Disease-free interval
ST	Survival time
HR	Harzad ratio
CI	Confidence interval
GTV	Gross tumor volume
CTV	Clinical target volume
OAR	Organs at risk
3D-CRT	Three-dimensional conformal radiotherapy
IMRT	Intensity-modulated radiotherapy
SRT	Stereotactic radiation therapy
VRTOG	Veterinary Radiation Therapy Oncology Group
ACVR	American College of Veterinary Radiology
ECVDI	European College of Veterinary Diagnostic Imaging
cRECIST	Veterinary Cooperative Oncology Group response evaluation
CNS	Central nervous system
